# Transcatheter Atrial Septal Defect Closure in Children with and without Fluoroscopy: A Comparison

**DOI:** 10.1155/2019/6598637

**Published:** 2019-04-07

**Authors:** S. Ackermann, D. Quandt, N. Hagenbuch, O. Niesse, M. Christmann, W. Knirsch, O. Kretschmar

**Affiliations:** ^1^Pediatric Cardiology, Pediatric Heart Center, Department of Surgery, University Children's Hospital Zurich, Switzerland; ^2^Children's Research Centre, Zurich, Switzerland; ^3^University of Zurich, Epidemiology, Biostatistics and Prevention Institute, Zurich, Switzerland

## Abstract

**Objective:**

The aim of this study was to compare feasibility, effectiveness, safety, and outcome of atrial septal defect (ASD) device closure in children with and without fluoroscopy guidance.

**Methods and Results:**

Children undergoing transcatheter ASD closure between 2002 and 2016 were included into this single center, retrospective study. Patients were analysed in two groups [1: intraprocedural fluoroscopy ± transoesophageal echocardiography (TOE) guidance; 2: TOE guidance alone]. Three-hundred-ninety-seven children were included, 238 (97 male) in group 1 and 159 (56 male) in group 2. Two-hundred-twenty-nine of 238 (96%) patients underwent successful fluoroscopy guided ASD closures versus 154/159 (97%) successful procedures with TOE guidance alone. Median weight (IQR) at intervention was 20kg (16.0-35.0) in group 1 versus 19.3kg (16.0-31.2) in group 2. Mean (SD) preinterventional ASD diameter was 12.4mm (4.4) in group 1 versus 12.2mm (3.9) in group 2. There was no significant difference in number of defects or characteristics of ASD rims. Median procedure time was shorter in group 2 [60min (47-86) versus 34min (28-44)]. Device-size-to-defect-ratio was similar in both groups [group 1: 1.07 versus group 2: 1.09]. There were less technical intraprocedural events in group 2 [10 (6.3%) versus 47 (20%)]. Intraprocedural complications were less frequent in group 2 [1 (0.6%) versus 8 (3.3%)].

**Conclusion:**

Transcatheter ASD device closure with TOE guidance alone (i.e., without fluoroscopy) is as effective and safe as ASD closure with fluoroscopy guidance. As fluoroscopy remains an important adjunct to transoesophageal echocardiography, especially in complex defects and complications, procedures are always performed in a fully equipped cardiac catheterization laboratory.

## 1. Introduction

Atrial septal defect (ASD) is one of the most common congenital heart diseases. Its incidence is reported at approximately 1.6 per 1000 live births [1]. In the 1980s the predominately surgical closure of ASDs was revolutionized by the introduction of umbrella-like devices delivered through cardiac catheters for interventional ASD device closure [2–5]. Fluoroscopy has emerged as gold standard imaging technique for catheter manipulation, guidewire control, balloon measurement of ASD diameter, and correct device placement throughout the procedure.

Nowadays the disadvantages of radiation exposure for the patient and for the catheterization laboratory staff in general with risk for development of cataract, arteriosclerosis, and especially cancer are well known [6–16]. Especially in children exposure to radiation and fluoroscopy should be kept as low as possible due to their high radiosensitivity and their large number of expected life years in which radiation related side effects could potentially occur [13].

Imaging accuracy of periprocedural 2D (and 3D) transthoracic and transoesophageal echocardiography (e.g., defect anatomy, defect size and rims, device and material visualisation) improved over the decades. Furthermore, better understanding of the haemodynamic relevance of atrial shunts determined by imaging techniques has arisen over the years [17]. This led to a “simplification” of the procedure, where full haemodynamic assessment, oximetry, and calculation of the Qp/Qs ratio of pulmonary blood flow (Qp) to systemic blood flow (Qs) was mostly given up. The indication for ASD closure relies mainly on clinical and echocardiographic features [18]. Some centers nowadays use transoesophageal echocardiography (TOE) guidance alone as imaging technique during interventional ASD device closure procedures. The feasibility of an exclusively TOE guided ASD device closure procedure is well described in the literature [19–22].

However, ASD device closure without the use of fluoroscopy has not been established as standard procedure in most centers as yet. Missing are data comparing interventional ASD device closure procedures with and without the use of fluoroscopy.

This study therefore set out to analyse and compare the effectiveness, safety, and outcome of interventional ASD device closure in a large group of children with and without the use of fluoroscopy. Furthermore, we will discuss the impact of balloon sizing, technical problems with device implantation, and complications. We hypothesized that interventional ASD closure under TOE guidance alone is as successful and safe as under fluoroscopy guidance.

## 2. Methods

**Figure 1:**
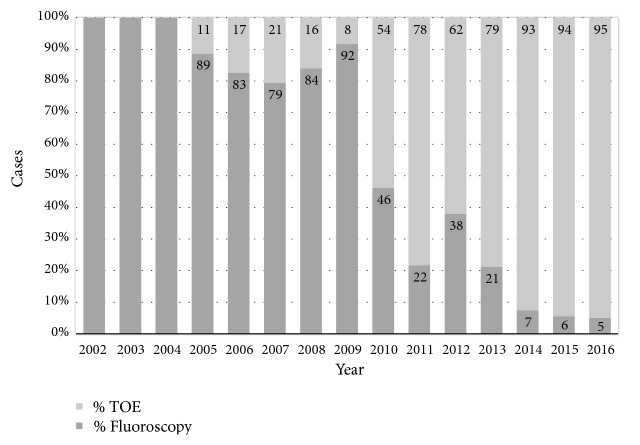
Development of the proportion of interventions performed with TOE guidance only or under fluoroscopy guidance in relation to all cases per year. Significant increase of TOE only guided interventional ASD device closures over the last years. TOE = transoesophageal echocardiography.

This study was performed as a retrospective, observational single center study at the University Children's Hospital Zurich in Switzerland. All consecutive children who underwent interventional, transcatheter ASD device closure between 2002 and 2016 (15 years) were included. Whether the intervention was preformed using fluoroscopy guidance or TOE guidance alone was a result of improved echocardiographic imaging techniques, a simplification of the procedure itself and therefore changing conviction over time. Therefore, almost all interventional ASD device closures in the recent era were performed using almost exclusively TOE guidance, which was primarily reserved for patients with single defects. Patients with multiple defects were primarily planned for ASD device closure using additional fluoroscopy guidance. Most of the fluoroscopy guided interventions were performed in the era between 2002 and 2009 (also see Figure 1).

The following exclusion criteria were applied: primarily planned additional cardiac catheter intervention other than ASD device closure, acute vasoreactivity testing (AVT) in patients with pulmonary hypertension or missing patients' or parental consent for further research data analysis.

General patients' demographics were analysed. Preinterventional two-dimensional transthoracic echocardiography (TTE) examinations were reviewed regarding number of ASDs, ASD diameter (mm), and structure of the ASD rim. The rim was defined as short if measured <5mm in at least one direction or as sufficient when measured ≥5mm or described as being “sufficient” for interventional device closure.

**Figure 2:**
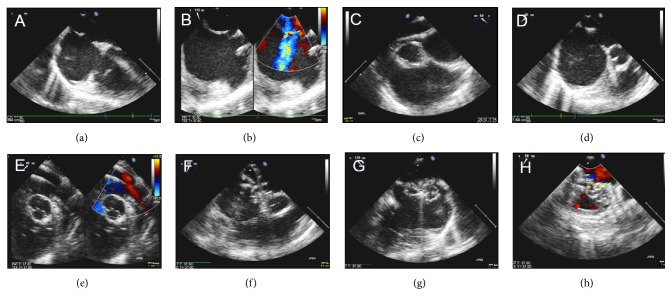
Illustration of interventional ASD device closure with a GORE Cardioform Septal Occluder using exclusively transesophageal echocardiography guidance. Standardized “step-by-step” procedural imaging guidance. ((a)+(b)) Imaging of the native defect, screening the defect and its rims from 0°-120°, focusing on standardized views in approximately 0°, 30°, 60°, 90°, and 120°. (c) Guidance of catheter passage across the defect into the left upper pulmonary vein (LuPV). (d) “Stretched” ASD diameter after stiff guide wire placement through the catheter into LuPV. (e) Illustration of large sheath insertion into LuPV. (f) Guidance of device deployment into the defect. ((g)+(h)) Illustration of device position after implantation in different views, again screening the defect and device from 0°-120°, focusing on standardized views in approximately 0°, 30°, 60°, 90°, and 120°.

The procedural steps for ASD device closure, including its echocardiographic imaging views, were highly standardized at our institution to optimize tracking of catheters, sheaths, and devices during the procedure, regardless of the device type used for implantation (Figure 2). Four different first operators performed 91% of all interventions.

Procedure related data were collected such as procedure time (min), dose area product (Gycm^2^), fluoroscopy time (min), ASD diameter at intervention (mm), ASD measurement technique, and implanted device type. The following technical events during the procedure were analysed: (1) having more than one device implanted, (2) choosing an improper device size, so that the device had to be explanted and changed (missizing), (3) need for device repositioning, and (4) equipment failure. Besides technical aspects major complications during the intervention were analysed and categorized: (1) device embolization and (2) heart rhythm disturbance. For calculation of the ratio of ASD diameter to the device size implanted, only self-centering devices as the Amplatzer™ Septal Occluder (AGA Medical, Golden Valley, MN, USA), NitOcclud® ASD-R-Device (PFM Medical AG, Cologne, Germany) and CeraFlex™ ASD Occluder (Lifetech scientific, Shenzhen, China) were analysed to overcome bias due to fixed device diameters of other devices used [e.g., Solysafe™ Septal Occluder (Swissimplant AG, Solothurn, Switzerland), GORE™ CARDIOFORM Septal Occluder (GORE Medical, Flagstaff, AZ, USA)].

Data of postinterventional echocardiograms were reviewed for residual atrial shunt, patients' weight and height. In addition, diameters of the right and left end diastolic ventricular diameter (RVEDD and LVEDD) in (mm) were reviewed and transformed to z-scores using the Echo z-Score Calculator developed at the Children's Hospital of Michigan (Detroit Data: http://parameterz.blogspot.ch/2008/09/z-scores-of-cardiac-structures.html) [23].

**Figure 3:**
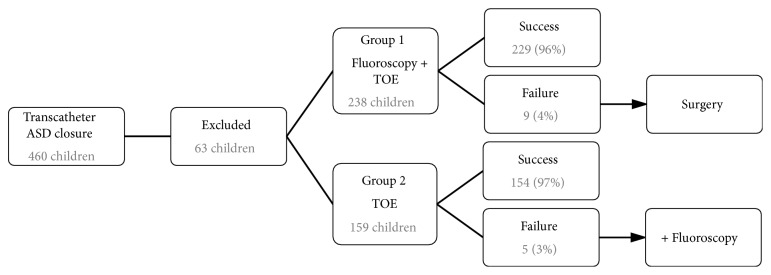
Patients flow chart showing all n=460 consecutive patients undergoing interventional ASD device closure between 2002 and 2016 and their distribution to the fluoroscopy group (group 1) and TOE alone group (group 2), as well as success and failure rates.

For statistical analysis, patients' personal data were anonymized and divided into two groups. Group 1 consisted of all children with an ASD device closure with fluoroscopy and optional TOE guidance and group 2 included all children with an ASD device closure with TOE guidance alone (Figure 3). Successful ASD closure was defined as correct device implantation performed under fluoroscopy and TOE guidance in group 1 or under TOE guidance alone in group 2. Statistical calculations were performed using SPSS (IBM SPSS Statistics version 23). Normally distributed data were presented as mean and standard deviation (SD), data of other distributions as median and interquartile range (IQR). Group differences regarding continuous variables were assessed using independent-sample t-test or Mann-Whitney-U test, and differences in proportions were analysed using the chi^2^ test or Fisher's exact test. Statistical significance was set at p<0.05. The study was performed in accordance with local ethic guidelines and was approved by the local ethics committee.

## 3. Results

### 3.1. Patient Characteristics

Between 2002 and 2016 a total of 460 children underwent interventional ASD device closure at our institution. An average of approximately 25-30 interventional ASD device closures were performed per year. The number of TOE only guided ASD device closures increased since 2002 from 0% to 95% in 2016 (Figure 1). Due to exclusion criteria met, 63 children had to be excluded from further analysis. The number of children included in the final analysis with a fluoroscopy guided intervention was 238 (group 1) versus 159 children, who underwent an exclusively TOE guided intervention (group 2).

Overall, the study population included 13 (3%) preterm infants, 24 (6%) with a defined genetic syndrome, and 58 (15%) with an additional cardiac diagnosis. Preinterventional suspicion of mild pulmonary hypertension on echocardiography was present in 104 (26%) cases. Basic characteristics for both groups are detailed in Table 1. There were no significant preinterventional group differences.

### 3.2. Procedural Findings

Intraprocedural measurements of ASD diameters in group 1 (fluoroscopy group) were performed using TOE measurement of native ASD diameter alone in 52 (22%) patients, balloon sizing of the defect in 164 (70%), and guidewire-stretched ASD diameters measured on TOE in 20 (8%) patients. In group 2 (exclusively TOE guided intervention), in 58 (36.5%) patients the native ASD diameter was measured, in 16 (10%) procedures balloon sizing was performed, and in 85 (53.5%) patients guidewire-stretched ASD diameters were assessed on TOE (p <0.001, chi^2^ test). The mean intraprocedural ASD diameter was 13.5 mm (SD 5.0) in group 1 versus 12.3 mm (4.0) in group 2 (p = 0.012, t-test). The Amplatzer™ Septal Occluder (AGA Medical, Golden Valley, MN, USA) was the device most frequently implanted. More details on the type of devices used are shown in Table 2.

The mean diameter of implanted devices in group 1 was 15.1 mm (SD 5.0) versus 13.6 mm in group 2 (4.2; p=0.003). There was no significant difference in device-size-to-defect-ratio between the groups [group 1: 1.07 mm (1.00-1.13); group 2: 1.09 mm (IQR 1.04-1.14); p = 0.133, Mann-Whitney-U test]. Median fluoroscopy time in group 1 was 9.3 min (6–16), and median dose area product was 3.4 Gycm^2^ (2-9). Median procedure time was 60 min (47–87) in group 1 versus 34 min (28–44; p <0.001, Mann-Whitney-U test) in group 2. Important technical procedure related events occurred in 20% of all cases in group 1 versus 6% in group 2 (p <0.001, chi^2^ test). For more details see Table 2.

Overall, most of the transcatheter ASD device closures were successful in both groups (96% versus 97%, p=0.736, chi^2^ test). Two-hundred-twenty-nine of 238 cases (96%) were closed successfully under fluoroscopy guidance. In the remaining 9 “unsuccessful” cases, surgery was successfully performed. In 154 of 159 cases (97%), ASDs were closed successfully under TOE guidance alone. In 4/5 of the remaining primarily unsuccessful cases, additional fluoroscopy guidance was used secondarily to successfully perform interventional ASD device closure. Secondary fluoroscopy was installed in 2 patients with device embolization (Solysafe™ septal occluder n=1, GORE™ CARDIOFORM septal occluder n=1), 1 patient with additional ASDs and necessity for multiple device implantations and 1 patients with attempted implantation of a GORE™ CARDIOFORM septal occluder to improve visibility of this device type. One child in group 2 had to be relocated to surgical ASD closure due to device embolization.

Intraprocedural complications are shown in Table 2. They occurred in 8 (3.3%) cases in group 1. There were 2 intraprocedural device embolizations in group 1. In 6 cases (Amplatzer™ Septal Occluder n=5, Solysafe™ Septal Occluder n=1) transient heart rhythm disturbances (e.g., transient AV-dissociation, transient AV-Block or nonsustained SVT) occurred. In group 2, there was one case (0.6%) of a male patient with deficient retroaortic ASD rim in which the device (Amplatzer™ Septal Occluder) embolized.

### 3.3. Postprocedural Follow-Up

Complications within 24 hours after intervention were detected in 8 cases (3.2%) of group 1 and in 4 cases (2.5%) of group 2. There was one device embolization within 24 hours in each group.

Device embolization affected two male children aged 6 and 7 years, both with a large ASD (18mm) with respect to their age and bodyweight (defect/bodyweight ratio 0.84, 1.05 respectively). In both cases an Amplatzer™ Septal Occluder (18mm) had been used and device retrieval was performed surgically (n=1) or interventionally (n=1). Details of further complications within 24 hours are shown in Table 2.

A total of 10 cases were registered with complications within 30 days. 7 complications occurred in group 1: 2 cases of thrombus formation, 1 case with mild regurgitation of the mitral valve, one case with transient AV-Block, and one with an equipment failure (device wire fracture of a Solysafe™ Septal Occluder). In addition, there was one case with an erosive fistula from left atrium to the aorta and one case with an erosion of the ascending aorta. Both of them had to be operated.

In group 2, there was one case of a male patient with thrombosis of the right iliac artery and another patient suffering from temporary palpitations.

At 3-month follow-up, there were no significant differences in the proportion of patients with residual atrial shunt on echocardiography imaging [group 1: 29 (15%), group 2: 25 (18%)]. One year after intervention, the percentage of children with residual shunt decreased to 11% in group 1 and to 13% in group 2, respectively. All of these residual shunts were haemodynamically not relevant.

**Figure 4:**
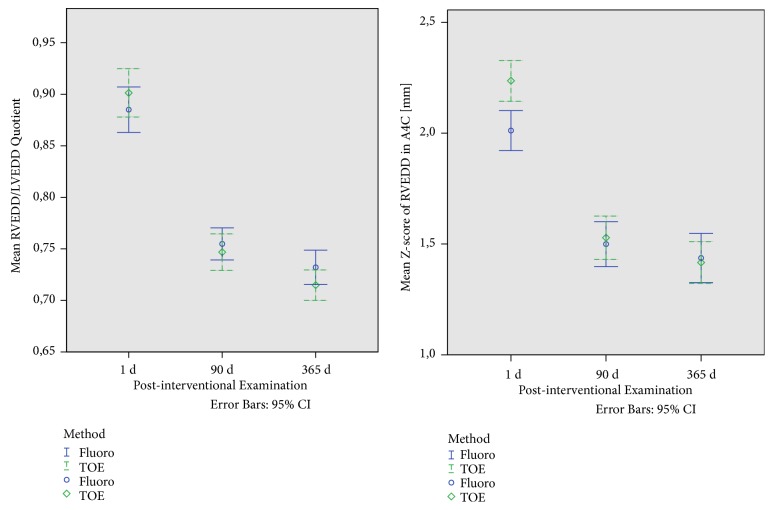
Postinterventional adaption of the RVEDD (z-score; right) and ratio of RVEDD to LVEDD (left) 1 day, 3 months and 1 year after intervention in the fluoroscopy group (blue line) and in the TOE alone group (green line). No relevant differences exist between the two groups.

The mean z-score of the RVEDD measured by TTE before the intervention was 2.2 (0.8) in group 1 and 2.4 (0.6) in group 2. The adaption and normalization of RVEDD z-scores and of the ratio of RVEDD to LVEDD after the intervention is shown in Figure 4 for both groups.

## 4. Discussion

Interventional transcatheter ASD device closure is recommended as treatment of first choice in grown-ups with congenital heart disease (GUCH) patients and children with hemodynamic relevant ASD by the European Society of Cardiology [24]. This is in line with the American College of Cardiology Foundation and American Heart Association stating that the majority of ASDs can be closed by percutaneous catheter techniques [25]. A statement about the favorable imaging technique used to guide the procedure is lacking. In literature, echocardiographic imaging is generally named as additional imaging technique besides fluoroscopy to evaluate ASD anatomy [26]. Ewert et al. had first published results on the feasibility of interventional ASD device closure without the use of fluoroscopy in a total of 26 patients in 1999 and 2000 [21, 22]. Schubert et al. assessed the same technique in 330 patients and published their results in 2012 [20]. In 2016, Yang et al. published results of 114 children who underwent transcatheter device closure under TOE guidance without fluoroscopy [19]. Yang emphasizes the fact that this modified technique has not been accepted as standard method yet, although the group showed a success rate of 96.5%, which is comparable to the results of Ewert et al. (23/26, success rate 88.5%) and Schubert et al. (success rate 98%), as well as results of ASD device closures performed under fluoroscopy guidance (success rate 96%) by Everett et al. [27].

Our report is the largest comparative study in children (n=397 children included in final analysis) on ASD device closure in children with and without fluoroscopy. Our study showed a success rate of 97% in transcatheter ASD device closure using exclusively TOE guidance. Most important, the success rate of TOE guided interventions did not differ from the success rate of interventions performed under fluoroscopy guidance. Risk factors for nonsuccessful interventional ASD closures were examined in a study of Mulukutla et al. published recently [28]. Patients with a high ratio of defect size to body surface area (ASD index) or a deficient rim were most likely to fail in interventional ASD closure, particularly when these factors were present in combination. Our results are consistent with these findings, as all of the patients with failed interventions of group 1 had either an ASD index above the average and/or deficient ASD rim (group 1: 4 cases with deficient RIM, 1 case with large ASD, 4 cases combination of both). This also applies to patients from group 2, where failing did not lead to surgery but to secondary use of fluoroscopy. Additional fluoroscopy was used to implant an additional device, supporting secondary correct device placement or the assessment whether the device was correctly positioned.

Regarding the best technique of correct measurement for ASD diameter sizing, this remains controversial. Balloon sizing is the standard procedure in most institutions and can also be done under TOE guidance (“stop-flow-technique”). In the instructions for the use of the most popular Amplatzer™ Septal Occluder [29], the user is cautioned not to overinflate the balloon in order to avoid oversizing. Other studies stress this fact [30, 31]. Balloon oversizing correlates directly with the use of larger devices. In our study, no significant group differences in preinterventional ASD diameters measured by TTE were observed. However, there was a significant difference in the rate of balloon sized ASDs between group 1 and 2. There was a greater number of patients undergoing balloon sizing under fluoroscopy guidance (group 1) in our cohort, accordingly significant larger ASD diameters were measured intraprocedural in group 1. Not the use of fluoroscopy, but rather the balloon-sizing technique itself, might be an important attribute for defect oversizing. Consequently, larger devices were implanted.

We found shorter procedure times in exclusively TOE guided interventions. This finding is biased by the fact that most of these interventions using TOE guidance alone were performed in the later period. Multiple different reasons have potentially influenced these shorter procedure times, e.g., less performance of haemodynamic assessment, less balloon sizing, use of multiple different devices, and an increasing operator experience over time. Causal for the greatest part of this reduction in procedure time might be the fact that in standard cases (i.e., single ASD) no haemodynamic assessment (oximetry, calculation of Qp/Qs) was performed. As a matter of fact, the indication for ASD closure is based on preinterventional TTE measurements. Moreover, haemodynamic assessment (i.e., Qp/Qs calculation) generates no relevant additional information in most routine cases. Already in 1985, Shub et al. proposed to avoid cardiac catheterization before surgical ASD [32]. This group could not find a difference in operative deaths or perioperative complications. Ewert et al. also questioned haemodynamic assessment in uncomplicated cases [21] and reliance on clinical and echocardiography assessment is well described in literature [33, 34].

Obviously, TOE guided interventions have the advantage of lacking radiation exposure. In our study the median radiation exposure in the fluoroscopy guided ASD device closure group was 3.4 Gy x cm^2^. With the use of new fluoroscopy imaging systems, radiation exposure can be reduced even if there were no other changes in interventional practice. Haas et al. studied a new X-ray imaging technology (Philips AlluraClarity, which is nowadays also used in our institution) and showed that there is a reduction of radiation dosage of 56% or more, especially in interventional ASD closures [35]. Further modifications should be evaluated and considered by the operator during the performance of interventional work (e.g., necessity for hemodynamic assessment and/or balloon sizing, use of low frame rates, limited cine acquisition, and use of single projection) to further reduce radiation exposure in general.

## 5. Limitations

In this retrospective, single center study no randomization was performed. Measurement techniques as they were performed on echocardiographic examinations are not standardized and might vary between cardiologists. The retrospective study design covers a period of 15 years. Over the years, also the operators' experience increased, which might have influence procedure times as operators become technically better and faster. The growing interventional skills, the availability of new device types, and the standardization of the procedure for the operators introduce some bias and might be an additional reason for less technical intraprocedural events and shorter procedure times assessed in group 2.

Although this is a large patient collective, the number of failed interventions in this population is small. Therefore, further (multicenter) studies and registries with adequate numbers of patients are necessary to analyse aspects that influence the rate of success and potential risk factors for unsuccessful device closure, device embolization, or erosion in detail.

## 6. Conclusion

Transcatheter ASD device closure with TOE guidance alone (i.e., without fluoroscopy) is as effective and safe as interventional ASD closure with fluoroscopy guidance. Key factors for a successful, exclusively TOE guided ASD device closure are standardized procedures, including periprocedural echocardiographic guidance. The experience of the operator, regarding assessment of the defect with its marginal structures, the behavior of the implant with and without the guidewire, or the relationship of the implant to neighboring intracardiac structures are further important factors for success. As fluoroscopy remains an important adjunct to transoesophageal echocardiography, especially in complex defects and complications, procedures are always performed in a fully equipped cardiac catheterization laboratory.

## Figures and Tables

**Table 1:** Patients demographics and characteristics in the fluoroscopy group (n=238) and the TOE group (n=159). Preinterventionally, there were no significant group differences. BSA = body surface area, ASD = atrial septal defect, IQR = interquartile range.

Variables	Fluoroscopy Group	TOE Group	p value
*Patient Characteristics*			

Male, n (%)	97 (41%)	56 (35%)	p = 0.267

Age [y], median (IQR)	6.1 (3.8-10.6)	5.7 (4.1-9.6)	p = 0.632

Weight [kg], median (IQR)	20 (16-35)	19 (16-31)	p = 0.745

*Number of ASDs*			
One, n (%)	180 (76%)	129 (81%)	p = 0.070
Two, n (%)	26 (11%)	7 (4%)	
Multiperforated, n (%)	32 (13%)	23 (15%)	

*ASD Rim*			
Short/deficient, n (%)	86 (43%)	74 (49%)	p = 0.263
Sufficient, n (%)	114 (57%)	77 (51%)	

Preintervention ASD diameter [mm], mean (SD)	12.4 (4.4)	12.2 (3.9)	p = 0.564

Intraprocedural ASD diameter [mm], mean (SD)	13.5 (5.0)	12.3 (4.0)	p = 0.012

**Table 2:** Illustration of different device types used for interventional ASD device closure in both groups, technically challenging or special events occurring during cardiac catheter device closure procedures in both groups [significantly more events in the fluoroscopy group (p<0.001)] and illustration of intraprocedural complications and complications within 24 hours after procedure in both groups.

Variables	Fluoroscopy Group	TOE Group
	(n = 238)	(n = 159)
*Device Types*		

Amplatzer™ Septal Occluder, n (%)	167 (72%)	125 (80%)
Solysafe™ Septal Occluder, n (%)	45 (20%)	0
CeraFlex™ ASD Occluder, n (%)	0	11 (7%)
GORE™ CARDIOFORM Septal Occluder, n (%)	4 (2%)	9 (6%)
pfm NitOcclud® ASD-R-Device, n (%)	5 (2%)	13 (8%)
BioSTAR™ Device, n (%)	8 (4%)	0
HELEX™ Septal Occluder, n (%)	3 (1%)	0

*Technical Aspects*		

No event, n (%)	191 (80%)	149 (94%)
Two devices needed, n (%)	13 (5%)	0
Three devices needed, n (%)	2 (1%)	0
Missizing, n (%)	13 (6%)	5 (3%)
Device repositioning, n (%)	19 (8%)	4 (3%)
Equipment failure, n (%)	0	1 (1%)

*Intraprocedural Complications*		

No complication, n (%)	230 (96.7%)	158 (99.4%)
Device embolization, n (%)	2 (0.8%)	1 (0.6%)
Heart rhythm disturbance, n (%)	6 (2.5%)	0

*Postprocedural Complications within 24 hours*		

No complication, n (%)	230 (96.6%)	155 (97.5%)
Device embolization, n (%)	1 (0.4%)	1 (0.6%)
Heart rhythm disturbance, n (%)	2 (0.8%)	2 (1.3%)
Pericardial effusion, n (%)	2 (0.8%)	0
Vascular access related problems, n (%)	1 (0.4%)	0
Impairment of neighbouring cardiac structures, n (%)	2 (0.8%)	1 (0.6%)

## Data Availability

Data were extracted from medical records in the Pediatric Cardiology Unit, Pediatric Heart Center, Department of Surgery, University Children's Hospital Zurich.

## Abbreviations

ASD: Atrial septal defect
AVT: Acute vasoreactivity testing
IQR: Interquartile range
LVEDD: Left ventricular enddiastolic dimension diameter
Qp: Pulmonary blood flow
Qs: Systemic blood flow
RVEDD: Right ventricular enddiastolic dimension diameter
SD: Standard deviation
TOE: Transoesophageal echocardiography.
